# Antimicrobial susceptibility/resistance and NG-MAST characterisation of *Neisseria gonorrhoeae* in Belarus, Eastern Europe, 2010–2013

**DOI:** 10.1186/s12879-015-0755-9

**Published:** 2015-01-31

**Authors:** Fiodar Lebedzeu, Daniel Golparian, Leonid Titov, Nataliya Pankratava, Slavyana Glazkova, Irina Shimanskaya, Natallia Charniakova, Aliaksandr Lukyanau, Marius Domeika, Magnus Unemo

**Affiliations:** The Republican Research and Practical Center for Epidemiology and Microbiology (RRPCEM), Minsk, Belarus; WHO Collaborating Centre for Gonorrhoea and other Sexually Transmitted Infections, Swedish Reference Laboratory for Pathogenic Neisseria, Department of Laboratory Medicine, Microbiology, Örebro University Hospital, Örebro, Sweden; Mogilev Regional Dermato-Venerological Dispensary, Mogilev, Belarus; Minsk City Dermato-Venerological Dispensary, Minsk, Belarus; Vitebsk Regional Dermato-Venerological Dispensary, Vitebsk, Belarus; Department of Prevention and Control of Communicable Diseases, Uppsala County Council, Uppsala, Sweden

**Keywords:** *Neisseria gonorrhoeae*, Gonorrhoea, Antimicrobial resistance, Surveillance, Extended-spectrum cephalosporins (ESCs), Ceftriaxone, Cefixime, Treatment, *N. gonorrhoeae* multiantigen sequence typing (NG-MAST), Belarus

## Abstract

**Background:**

Gonorrhoea and widely spread antimicrobial resistance (AMR) in its etiological agent *Neisseria gonorrhoeae* are major public health concerns worldwide. Gonococcal AMR surveillance nationally and internationally, to identify emerging resistance and inform treatment guidelines, is imperative for public health purposes. In 2009, AMR surveillance was initiated in Belarus, Eastern Europe because no gonococcal AMR data had been available for at least two decades. Herein, the prevalence and trends of gonococcal AMR and molecular epidemiological characteristics of *N. gonorrhoeae* strains from 2010 to 2013 in Belarus, are described.

**Methods:**

*N. gonorrhoeae* isolates (n=193) obtained in the Mogilev (n=142), Minsk (n=36) and Vitebsk (n=15) regions of Belarus in 2010 (n=72), 2011 (n=6), 2012 (n=75) and 2013 (n=40) were analyzed in regards to AMR using the Etest method and for molecular epidemiology with *N. gonorrhoeae* multi-antigen sequence typing (NG-MAST).

**Results:**

During 2010–2013, the proportions of resistant *N. gonorrhoeae* isolates were as follows: tetracycline 36%, ciprofloxacin 28%, penicillin G 9%, azithromycin 5%, and cefixime 0.5%. Only one (0.5%) β-lactamase producing isolate was detected. No isolates resistant to ceftriaxone and spectinomycin were identified. Overall, the resistance levels to tetracycline, ciprofloxacin and penicillin G were relatively stable. Interestingly, the level of resistance to azithromycin declined from 12% in 2010 to 0% in 2013 (P < 0.05). In total, 70 NG-MAST STs were identified. The predominant STs were ST1993 (n=53), ST807 (n=13), ST285 (n=8) and ST9735 (n=8). Many novel STs (n=43, 61%), representing 41% of all isolates, were found.

**Conclusions:**

During 2010–2013, the *N. gonorrhoeae* population in Belarus displayed high and relatively stable resistance levels to tetracycline, ciprofloxacin, and penicillin G, while the resistance to azithromycin declined. One isolate was resistant to cefixime, but no resistance to ceftriaxone or spectinomycin was found. The results of the present surveillance initiated in 2009 were also used to replace penicillin G with ceftriaxone (1 g single dose intramuscularly) as the first-line drug for empiric treatment of gonorrhoea in the national treatment guidelines in Belarus in late 2009. It is essential to further strengthen the surveillance of gonococcal AMR and ideally survey also treatment failures and molecular epidemiological genotypes in Belarus.

## Background

Gonorrhoea, etiological agent *Neisseria gonorrhoeae*, is a public health concern globally. In most of the non-European Union (EU)/European Economic Area (EEA) countries (n=23), which mostly represent independent countries from the former Soviet Union and Yugoslavic Republic, of the World Health Organization (WHO) European Region (53 countries), the gonorrhoea incidence has rapidly declined during the last 20 years. In the Eastern European non-EU/EEA country Belarus (9.5 million inhabitants), the incidence has also mainly declined during the last two decades. However, the incidence remains high and in 2011 Belarus reported the third highest incidence (35.4 per 100 000 population) in the WHO European Region ([1,2],http://data.euro.who.int/cisid). The reported gonorrhoea incidences in Belarus might also be underestimated, which is due to suboptimal diagnostics, access to testing, case reporting, e.g. lack of reporting of cases diagnosed in the private sector, and surveillance [2-4].

*N. gonorrhoeae* has developed antimicrobial resistance (AMR) to all drugs previously recommended for treatment of gonorrhoea. *In vitro* (low-level and high-level) and rare treatment failures have now emerged to the last remaining option for first-line empirical monotherapy in most countries globally, that is, the extended-spectrum cephalosporin (ESC) ceftriaxone [5-18]. Therefore, enhanced and quality assured surveillance of gonococcal AMR, nationally and internationally, is essential to monitor the AMR trends, identify emerging AMR and inform a regular update of the STI management and treatment guidelines locally, nationally and globally. This is in strict concordance with the global action plan and European response plan to combat gonococcal AMR published by the WHO [19,20] and the European Centre for Disease Prevention and Control (ECDC) [21], respectively.

Worryingly, in the non-EU/EEA countries of the WHO European Region, quality assured gonococcal AMR surveillance only exist in 13% (3/23) of the countries [2]. In 2009, because no gonococcal AMR data had been available for at least two decades gonococcal AMR surveillance was initiated in Belarus by the Eastern European Network for Sexual and Reproductive Health [22] in collaboration with the WHO. This AMR surveillance has been quality assured in accordance with WHO standards and the 2008 WHO *N. gonorrhoeae* reference strains are used as quality controls [23].

The aims of the present study were to describe the prevalence and trends of gonococcal AMR and molecular epidemiological characteristics of *N. gonorrhoeae* strains from 2010 to 2013 in Belarus.

## Methods

### Study population

*N. gonorrhoeae* isolates (n=193) were obtained at the Mogilev Regional (n=142), Minsk City (n=36) and Vitebsk Regional (n=15) Dermato-Venerological Dispensaries in Belarus in 2010 (n=72), 2011 (n=6), 2012 (n=75) and 2013 (n=40). Mainly consecutive culture positive gonorrhoea patients from January 2010 to April 2013 were included. Urethral and cervical specimens from females and urethral specimens from males were collected.

All specimens were cultured on selective gonococcal agar media and suspected gonococcal colonies were subsequently confirmed as *N. gonorrhoeae* by colony morphology, Gram staining, oxidase test and a carbohydrate utilization test [24]. The isolates were stored in cryogenic storage dewar with liquid nitrogen (−196°C) or in ultra-low temperature freezer (−70°C) in preservation media and then shipped on dry ice to the WHO Collaborating Centre for Gonorrhoea and other STIs, Sweden for further analysis. All examined gonococcal isolates were cultured and stored as part of the routine diagnostics (standard care) and no patient identification information was available in the study.

### Antimicrobial susceptibility and β-lactamase testing

The minimum inhibitory concentration (MIC; mg/L) of ceftriaxone, cefixime, azithromycin, spectinomycin, ciprofloxacin, penicillin G, tetracycline and gentamicin was analysed using the Etest methodology on Difco GC Medium Base (Becton, Dickinson and Company, Sparks, MD, USA) supplemented with 1% BBL IsoVitaleX Enrichment (Becton, Dickinson and Company, Sparks, MD, USA), with agar plates incubated for 18–20 h at 35-37°C in a 5% CO_2_-enriched atmosphere, according to the instructions from the manufacturer (bioMérieux AB, Solna, Sweden). The Etest results were interpreted as susceptible (S), intermediate susceptible (I) and resistant (R) according to the breakpoints stated by The European Committee on Antimicrobial Susceptibility Testing (EUCAST; www.eucast.org/clinical_breakpoints). For gentamicin, no breakpoints are stated by any organization and, accordingly, only MIC range, MIC_50_ and MIC_90_ were used.

β-lactamase production was identified with nitrocefin solution (0.5 g/mL), according to the manufacturer’s instructions (Oxoid, Basingstoke, Hants, England).

### Isolation of genomic DNA

Genomic DNA was isolated using the NorDiag Bullet robot (NorDiag ASA Company, Oslo, Norway) with BUGS’n BEADS STI-fast kit (NorDiag ASA Company, Oslo, Norway), according to the manufacturer’s instructions.

### Molecular epidemiological typing

NG-MAST [25,26] was performed as previously described [27]. NG-MAST allele numbers of the more variable segments of *porB* and *tbpB*, and sequence types (STs) were assigned using the NG-MAST website (www.ng-mast.net).

### Statistical analysis

Statistical analysis was performed using the Statistica software version 9.0. Z-test for comparison of proportions was used. The level of significance was set at *P* < 0.05.

## Results

### Patient characteristics

*N. gonorrhoeae* isolates (one isolate per patient) from 193 patients; 169 (87.6%) males and 24 (12.4%) females, were examined. The mean age for the males was 26.9 years (median age: 25.0 years; range: 19 to 57 years) and for the females 27.3 years (median age: 28.5 years; range: 19 to 33 years). The male/female ratio and age distribution was relatively similar during the four years investigated.

### Antimicrobial susceptibility of *N. gonorrhoeae* isolates in 2010–2013 (n=193) in Belarus

The overall antimicrobial susceptibility of all isolates is summarized in Table 1.

**Table 1:** **Antimicrobial susceptibility of** ***Neisseria gonorrhoeae*** **isolates (n**=**193) from Belarus, 2010–2013**

**Antimicrobial**	**MIC**_**50**_ **/**	**Susceptible (%)**			**Intermediate susceptible (%)**			**Resistant (%)**		
	**MIC**_**90**_ **/**	**2010-2011**	**2012**	**2013**	**2010-2011**	**2012**	**2013**	**2010-2011**	**2012**	**2013**
	**MIC range**	**(n**=**78)** ^***a***^	**(n**=**75)**	**(n**=**40)**	**(n**=**78)** ^***a***^	**(n**=**75)**	**(n**=**40)**	**(n**=**78)** ^***a***^	**(n**=**75)**	**(n**=**40)**
	**(mg/L)**									
Penicillin G^*b*^	0.032/1/	-	88	78	-	5	10	-	7	13
	0.008-2									
Ceftriaxone^*b*^	0.008/0.047/	100	100	100	NA	NA	NA	0	0	0
	<0.002-0.125									
Cefixime^*b*^	0.023/0.047/	100	100	97.5	NA	NA	NA	0	0	2.5
	<0.016-0.25									
Azithromycin^*b*^	0.19/0.38/	65	83.3	90	23	15.3	10	12	1.3	0
	0.032-1									
Ciprofloxacin^*b*^	0.006/6/	64	79	72	1	0	0	35	21	28
	0.003- >32									
Tetracycline^*b*^	0.38/24/	56	56	55	8	9	5	36	35	40
	0.094-24									
Spectinomycin^*b*^	12/16/3-16	100	100	100	NA	NA	NA	0	0	0
Gentamicin^*c*^	2010-2011:				2012:			2013:		
	MIC range: 0.25-16 mg/L; MIC_50:_ 2 mg/L; MIC_90:_ 4 mg/L				MIC range: 2-16 mg/L; MIC_50:_ 4 mg/L; MIC_90:_ 8 mg/L			MIC range: 3-12 mg/L; MIC_50:_ 6 mg/L; MIC_90:_ 8 mg/L		

MIC_50_, minimum inhibitory concentration of 50% of isolates; MIC_90_, minimum inhibitory concentration of 90% of isolates; −, Not tested; NA, not applicable.

^*a*^Due to the few isolates obtained in 2011, isolates from 2010 (n=72) and 2011 (n=6) were evaluated as one time point.

^*b*^Breakpoints for susceptible and resistant isolates were according to the European Committee on Antimicrobial Susceptibility Testing (EUCAST; www.eucast.org/clinical_breakpoints).

^*c*^Breakpoints not stated by any organization.

Briefly, the levels of *in vitro* resistance during 2010–2013 were as follows: tetracycline 36%, ciprofloxacin 28%, penicillin G 9%, azithromycin 5%, and cefixime 0.5% (one isolates with an MIC of 0.25 mg/L). Only one (0.5%) β-lactamase producing isolate was found. No isolates resistant to ceftriaxone and spectinomycin were identified. Overall, the resistance levels to tetracycline, ciprofloxacin and penicillin G were relatively stable. Interestingly, the level of resistance to azithromycin significantly declined from 12% in 2010 to 0% in 2013 (P < 0.05). For gentamicin (MIC range: 0.25-16 mg/L), the MICs were relatively low and all isolates considered susceptible (Table 1).

**Figure 1:**
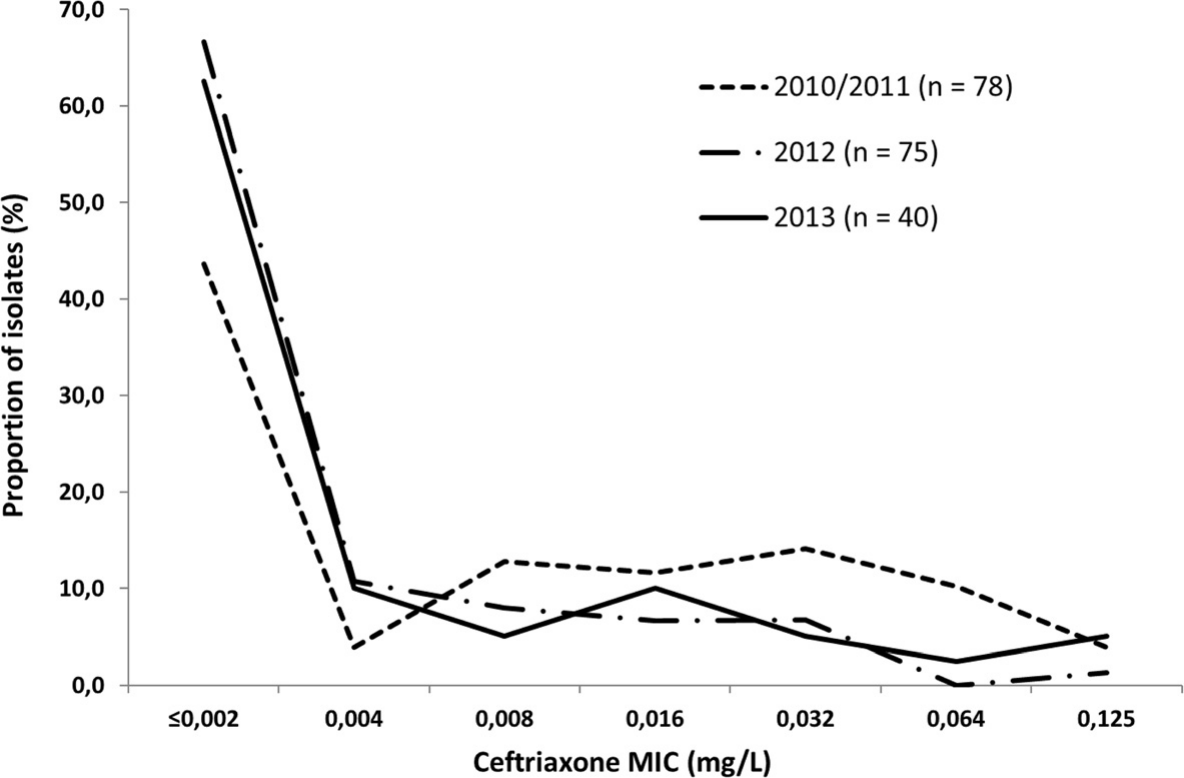
**The distribution of minimum inhibitory concentrations (MICs) of ceftriaxone for** ***Neisseria gonorrhoeae*** **isolates (n**=**193) cultured in Belarus from 2010 to 2013.**

The proportion of isolates with ceftriaxone MIC < 0.002 mg/L was 44%, 67%, and 63% in 2010/2011, 2012 and 2013, respectively, and in general the annual MIC distribution for ceftriaxone appeared to shift to lower MICs during the study period 2010–2013 (Figure 1). However, although no resistance to ceftriaxone was identified, in total 3.1% (5% in 2013) had a ceftriaxone MIC of 0.125 mg/L, which is exactly at the resistance breakpoint stated by the EUCAST (www.eucast.org/clinical_breakpoints). Gonococcal isolates with these MICs have previously resulted in ceftriaxone treatment failures and can be considered to have a decreased susceptibility [12-14,16-18].

### Molecular epidemiological typing

The 193 were assigned to 70 different NG-MAST STs. The most prevalent ST was ST1993 (n=53, 27.5% of isolates), followed by ST807 (n=13, 6.7%), ST285 (n=8, 4.1%), ST9735 (n=8, 4.1%) and ST2873 (n=6, 3.1%). The remaining STs were represented by five (1 cluster), four (n=3), three (n=8), two (n=11) and one (n=42) isolates. Forty-three (61%) of these STs were not previously described. In general, the most prevalent STs such as ST1993, ST807, ST285 and ST9735 had relatively low MICs of ceftriaxone. Notably, the cefixime resistant gonococcal isolate obtained in 2013 was assigned as ST3149.

## Discussion

In the present study, the AMR in *N. gonorrhoeae* isolates cultured from 2010 to 2013, and molecular epidemiological characteristics (NG-MAST) of *N. gonorrhoeae* isolates in Belarus were examined. Previously, only one minor AMR study examining gonococcal isolates from Belarus in 2009 [28] has been internationally published. Accordingly, exceedingly limited phenotypic and genetic characteristics of the gonococcal strains spreading in Belarus have been previously published.

High prevalences of resistance were observed for previously recommended antimicrobials such as tetracycline (36%), ciprofloxacin (28%), and penicillin G (9%). In accordance with the WHO recommendations regarding exclusion of an antimicrobial when the resistance level reaches ≥5% in the general population, none of these antimicrobials should be recommended for empirical first-line monotherapy of gonorrhoea in Belarus or in most other countries globally where resistance is prevalent [2,5-11]. In line with this, the results of the present surveillance initiated in 2009 [28] were also used to replace penicillin G with ceftriaxone as the first-line drug for empiric treatment of gonorrhoea in the national treatment guidelines in Belarus in late 2009 [29]. Interestingly, β-lactamase producing gonococcal strains appear to be exceedingly rare (1/193 isolates) in Belarus, which is also the case in some other independent countries of the former Soviet Union such as Russia [30-33]. In Belarus, where penicillin G and other penicillin derivatives have been widely used for treatment of gonorrhoea at least until 2010, this may indicate that no imported β-lactamase producing gonococcal strains have been established and resulted in an endemic spread in Belarus. Resistance to azithromycin was also relatively prevalent (5%), however, no isolates with high-level resistance to azithromycin (MIC ≥ 256 mg/L), which have been described from several other countries [34-39], have yet been identified in Belarus. Furthermore, the resistance to azithromycin significantly declined from 12% in 2010 to 0% in 2013, which might reflect that azithromycin is rarely used for treatment of gonorrhoea and utilized in a controlled manner in the treatment of other STIs such chlamydial infection.

Since late 2009, in Belarus ceftriaxone 1 g single dose intramuscularly is the nationally recommended first-line treatment for uncomplicated anogenital and extra-genital gonorrhoea [29]. In the present study, no ceftriaxone resistant isolates and only one (0.5%) cefixime resistant isolate were identified. This is in contrast to the findings in the neighbouring country Russia, where according to the EUCAST breakpoints (www.eucast.org) 2.7% of isolates were resistant to ceftriaxone in 2009–2012 [33]. The reasons for the low MICs of ceftriaxone in Belarus are unknown. However, this might reflect that no gonococcal strain with decreased susceptibility or resistance to ESCs has been imported and/or managed to establish any domestic spread. Furthermore, in Belarus cefixime or other less potent oral ESCs have never been widely used for treatment of gonorrhoea. No resistance to spectinomycin was either found. This is also in contrast to the findings in Russia, where isolates with low-level resistance to spectinomycin are detected every year [31-33]. Worryingly, in Belarus despite that ceftriaxone 1 g single dose intramuscularly is the recommended first-line regimen penicillins, fluoroquinolones, macrolides, and other cephalosporins remain to be used in the routine gonorrhoea treatment. Furthermore, antimicrobials are easily available “over-the-counter”, and this practice is crucial to abandon. The use of also less potent antimicrobials in the treatment of gonorrhoea, spread of gonococcal strains with decreased ESC susceptibility in Belarus and ESC resistance internationally, including in the neighbouring country Russia where also spectinomycin resistance are spreading, necessitate an enhanced surveillance of gonococcal AMR (with focus on ceftriaxone but also multidrug resistance), and ideally also treatment failures and molecular epidemiological characteristics in Belarus. There are also initiatives to improve the gonococcal AMR surveillance in Belarus, that is, to increase the number of isolates examined annually, the geographic representativeness by including additional regions, and the linkage to appropriate epidemiological data. In general, national and international support, including political and financial commitment, is essential to strengthen the gonococcal AMR surveillance in non-EU/EEA countries of the WHO European Region [2-4].

NG-MAST has been used in many countries worldwide; to monitor the national and international spread of gonococcal strains, including AMR clones, to identify transmission patterns within sexual networks, and to verify or falsify suspected treatment failures [26]. In Belarus, during 2010–2013 70 different NG-MAST STs among 193 isolates were identified. The substantial number of STs represented by only one isolate (n=42) and STs that have not been previously described (n=43; 61%) may be associated with the low number of cultured gonococcal isolates from each surveillance site, suboptimal diagnostics, lack of tracing of sexual contacts, and STs evolved locally in Belarus. Nevertheless, some major ST clusters of, e.g., ST1993 (n=53, 27.5% of isolates), followed by ST807 (n=13, 6.7%), ST285 (n=8, 4.1%), and ST9735 (n=8, 4.1%), were identified, which indicate some larger sexual transmission chains. No isolates assigned as ST1407, the internationally spread multidrug resistant gonococcal clone [5,8,11,14,18,40-44], was identified from 2010 to 2013 in Belarus.

## Conclusions

In Belarus, during 2010–2013 the heterogeneous gonococcal population showed high and relatively stable resistance levels to ciprofloxacin, tetracycline and penicillin G. The overall resistance to azithromycin was also relatively high, however, significantly declined from 2010 to 2013. One isolate was resistant to cefixime, but no resistance to ceftriaxone or spectinomycin was found. The results of the present surveillance initiated in 2009 [28] were also used to replace penicillin G with ceftriaxone (1 g single dose intramuscularly) as the first-line drug for empiric treatment of gonorrhoea in the national treatment guidelines in Belarus in late 2009. Accordingly, based on the present study and resistance data from worldwide [2,5-11,19,30-33,40-42,45], ceftriaxone should be the only option for first-line empiric antimicrobial monotherapy of gonorrhoea in Belarus. Spectinomycin should be the alternative treatment option and only used when ceftriaxone is not available or the patient suffers from a severe β-lactam allergy. However, if pharyngeal gonorrhoea has not been excluded azithromycin is recommended to be added to the spectinomycin regimen. It is essential to further strengthen the surveillance of gonococcal AMR and ideally survey also treatment failures and molecular epidemiological genotypes in Belarus.

## Abbreviations

AMR: Antimicrobial resistance
ECDC: European centre for disease prevention and control
EEA: European economic area
ESC: Extended-spectrum cephalosporins
EU: European union
EUCAST: European committee on antimicrobial susceptibility testing
MIC: Minimum inhibitory concentration
NG-MAST: *N. gonorrhoeae* multiantigen sequence typing
ST: Sequence type
WHO: World Health Organization
